# Unexpected Phase Behavior of Pluronic Polymer-Organic Derivative Mixtures Depending on Temperature in Aqueous Solution

**DOI:** 10.3390/mi9100505

**Published:** 2018-10-05

**Authors:** Jong-Dae Jang, Eunhye Kim, Min-Jae Lee, Young-Soo Han, Joona Bang, Tae-Hwan Kim

**Affiliations:** 1 Neutron Science Division, Research Reactor Utilization Department, Korea Atomic Energy Research Institute, 1045 Daedeok-daero, Yuseong-gu, Daejeon 34057, Korea; jdjang@kaeri.re.kr (J.-D.J.); eunhye20@jbnu.ac.kr (E.K.); yshan@kaeri.re.kr (Y.-S.H.); 2 Department of Chemical and Biological Engineering, Korea University, 145 Anam-ro, Sungbuk-gu, Seoul 02841, Korea; joona@korea.ac.kr; 3 Department of Quantum System Engineering, Chonbuk National University, 567 Baekje-daero, Deokjin-gu, Jeonju-si, Jeollabuk-do 54896, Korea; 4 Department of Nuclear and Quantum Engineering, Korea Advanced Institute of Science and Technology, 291 Daehak-ro, Yuseong-gu, Daejeon 34141, Korea; leekaist@kaist.ac.kr

**Keywords:** block copolymer, self-assembly, nanostructures, phase behavior, small angle neutron scattering (SANS)

## Abstract

The phase behavior of amphiphilic Pluronic block copolymers in aqueous solution is of importance for a broad spectrum of practical applications but has not been fully exploited yet. Here, the phase behavior of the mixture of the Pluronic P65 and P105 triblock copolymer, (which have the same composition of PEO and PPO but the different molecular weight) and organic derivative, 5-methyl salicylic acid (5mS), in aqueous solution has been investigated by using small angle neutron scattering (SANS). According to the temperature and the 5mS concentration, SANS measurements showed that the P65-5mS mixtures sequentially transform into a random coil, sphere, vesicle, cylinder, and vesicle again, while the P105-5mS mixtures form spherical particles with two different sizes without any topological phase transition. Upon heating, the formation of two different kinds of the vesicle structure of amphiphilic block copolymer in aqueous solution is very unusual. This phase behavior was explained as the coupled effect of the simultaneous increase of the hydrophobicity of the polymer and the solubility of 5mS molecules upon heating. This result gives fundamental information for the practical use of Pluronic polymers in nano- and bio-science and it provides a simple route for the fabrication of the nanostructure without a complicated procedure.

## 1. Introduction

Pluronic triblock copolymers (PEO_m_PPO_n_PEO_m_), which are one of representative amphiphilic molecules, have been of great interest for a large variety of phase behaviors and the self-assembly into various micellar structures, such as sphere, cylinder, or lamellae in aqueous solution. The self-assembled structure of Pluronic triblock copolymer is easily controlled according to the external solution conditions, such as temperature, additives, concentration, or pH [1,2,3,4,5,6,7]. Furthermore, the Pluronic triblock copolymers have the excellent ability for their property improvement through polymer engineering (i.e., Synthesis with a functional group). Therefore, it is expected to be a wide range of potential applications of Pluronic triblock copolymer in nano- or bio-science, such as nanobuilding blocks or drug delivery systems [8,9,10,11,12,13]. In spite of the extraordinary properties, however, there have been still many remaining problems (i.e., poor understanding for dynamic and structural properties [14], etc.) to be solved for practical application. One of key issues is to identify the phase behavior of Pluronic triblock copolymers under various external solution conditions that is the fundamental information for practical use and has not been fully examined yet, even though many efforts have been reported for this purpose [1,2,3,4,5,6,7].

It is well known that Pluronic triblock copolymers self-assemble into various micellar structures according to their molecular weight and composition ratio of PEO and PPO, which have different size, shape, critical micelle concentration (CMC), and temperature dependence in aqueous solution [14,15,16,17,18,19,20]. In this sense, when an additive (which can easily lead to the control of the effective geometrical molecular shape of block copolymers without their chemical synthesis) is mixed with the Pluronic triblock copolymers, therefore, it is expected that the phase behavior of the polymer-additive mixtures is also dependent with their molecular weight or composition ratio. However, the phase behavior of a mixture of Pluronic triblock copolymer with various molecular weights and composition ratios and additives has not been well known yet. Here, we report the phase behavior of the mixture of Pluronic P65 (PEO_20_PPO_30_PEO_20_, M_W_ = 3500 g/mol) and P105 (PEO_37_PPO_58_PEO_37_, M_W_ = 6500 g/mol) copolymers and an organic derivative, 5-methyl salicylic acid (5mS), as an additive, which has a strong tendency to bind with amphiphilic molecules that leads to various phase behaviors due to its temperature sensitive solubility and asymmetric amphiphilicity. Depending on the temperature and 5mS concentration, the P65-5mS mixture sequentially transforms into a random coil polymer, sphere, vesicle, cylinder, and vesicle again, while the P105-5mS mixture did not show any topological phase transition. Even though the polymeric vesicle structure was found in the previous study [8], the vesicle was not formed repeatedly upon heating. To the best of our knowledge, this is the first report of two times formation of vesicles in aqueous solution depending on temperature. Furthermore, we expect that the formation of vesicle structures in the specific temperature range can be applied to smart drug delivery systems for an organ with specific temperature, and this study can provide the fundamental information for practical use of Pluronic polymers into various building blocks as well as a simple way for their fabrication.

## 2. Experimental

### 2.1. Materials

Pluronic P65 (PEO_20_PPO_30_PEO_20_, M_W_ = 3500 g/mol), and P105 (PEO_37_PPO_58_PEO_37_, M_W_ = 6500 g/mol) triblock copolymers were purchased from BASF. 5-methyl salicylic acid (5mS) was purchased from Tokyo Chemical Industry Co., Ltd. (Tokyo, Japan). All of the chemicals were used as received without further purification. D_2_O (99.9 mol% deuterium enriched) was purchased from Cambridge Isotope Laboratory and H_2_O was purified by a Millipore Direct Q system immediately before use.

### 2.2. Sample Preparation

Pluronic triblock copolymers (P65 and P105) at the low concentration (0.25% by weight) were prepared in water. The organic derivative, 5mS, was varied from 0 to 0.125% and from 0 to 0.2% (by weight) for P65 and P105, respectively. To make a homogeneous mixture solution of Pluronic polymer and the organic derivative, we used a combination of the vortex mixing and the thermal agitating into 70 °C for 2 h. All of the mixture solutions were equilibrated for 1 day at 30 °C before all measurements.

### 2.3. SANS Measurements

SANS measurements were carried out the 40m SANS Instrument at HANARO in the Republic of Korea [21]. Neutrons with the wavelength of λ = 6 Å and full width half maximum Δλ/λ = 12% were used. Three different sample to detector distances (SDD = 1.16 m, 4.7 m, and 19.85 m) were used to cover the overall *q* range of 0.0016 Å^−1^ < *q* < 0.5 Å^−1^, where *q* is the magnitude of the scattering vector (*q = (4π/λ)sin(θ/2)*) and *θ* is the scattering angle. Sample scattering was corrected for background, empty cell scattering, and the sensitivity of individual detector pixels. The corrected data sets were placed on an absolute scale using the data reduction software provided by HANARO through the standard sample calibration method. To enhance the scattering contrast between solvent and samples, all of the SANS measurements were carried out in D_2_O.

### 2.4. SANS Analyses

To get a detailed structural information, all the SANS intensities was analyzed by non-linear least squares model fits using various numerical functions (sphere, core-shell sphere, core-shell cylinder, and vesicle) [22,23,24,25]. Since all of the mixture solutions were prepared at 0.25%, which is sufficiently dilute, the interparticle interference was not considered in the model fits. The used scattering length densities (SLDs) of Pluronic polymer [26,27], 5mS [28], and heavy water are 0.47 × 10^-6^ Å^−2^, 2.11 × 10^-6^ Å^−2^, and 6.4 × 10^-6^ Å^−2^, respectively.

## 3. Results and Discussion

Both Pluronic P65 and P105 triblock polymers have the mass composition of PEO and PPO with 1:1 (where the mass fraction of hydrophilic part *f* is 0.5), resulting in the formation of spherical or cylindrical micelles [1,2,3,4]. While considering the different molecular weight between Pluronic P65 and P105, however, its temperature dependent phase behavior is not identical, which means that Pluronic polymers can provide various self-assembled nanostructures depending on the temperature and molecular weight. Here, with controlling the mass composition of PEO and PPO, we expected that the Pluronic polymer can self-assemble into more various nanostructures. To achieve a variety of nanostructures of Pluronic polymer, therefore, we added the organic derivative, 5-methyl salicylic acid (5mS). The 5mS molecule consists of the salicylic acid with the methyl group (which is located at the 5-position) and it is slightly soluble in aqueous solution. Therefore, it strongly leads to the penetration of methyl groups together with the phenyl groups in the hydrophobic part of block copolymer, whereas the hydroxyl and carboxyl groups at the opposite site of the methyl group make contact with water. This makes a strong tendency to bind with the amphiphilic molecules [29,30], which provides a chance to get a new phase behavior. Then, the phase behavior of Pluronic P65 and P105 has been investigated with increasing the concentration of 5mS molecules.

**Figure 1:**
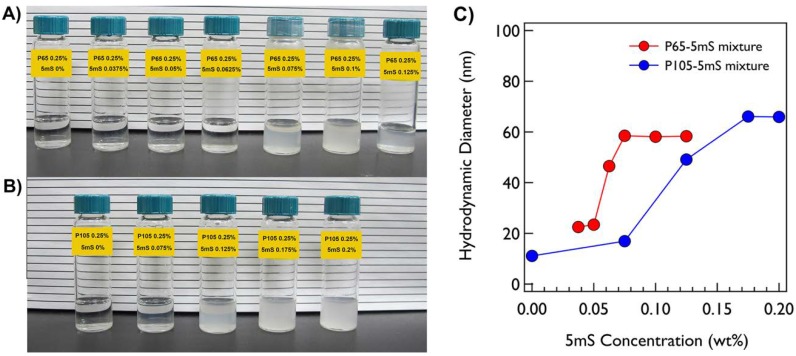
(**A**) Photos of P65-5mS mixtures at 25 °C. (**B**) Photos of P105-5mS mixtures 25 °C. (**C**) Hydrodynamic Diameters of P65-5mS and P105-5mS mixtures at 25 °C.

**Figure 2:**
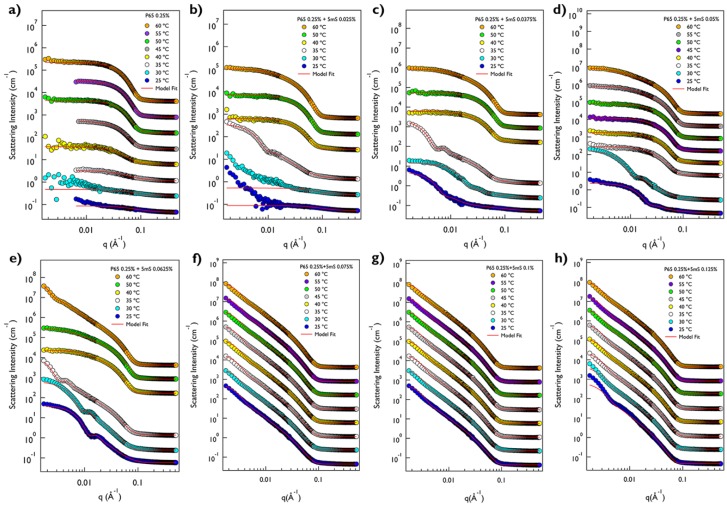
SANS intensities of P65-5mS mixtures in heavy water at varying 5mS concentration (**a**) 0%, (**b**) 0.025%, (**c**) 0.0375%, (**d**) 0.05%, (**e**) 0.0625%, (**f**) 0.075%, (**g**) 0.1%, and (**h**) 0.125% and temperature.

**Figure 3:**
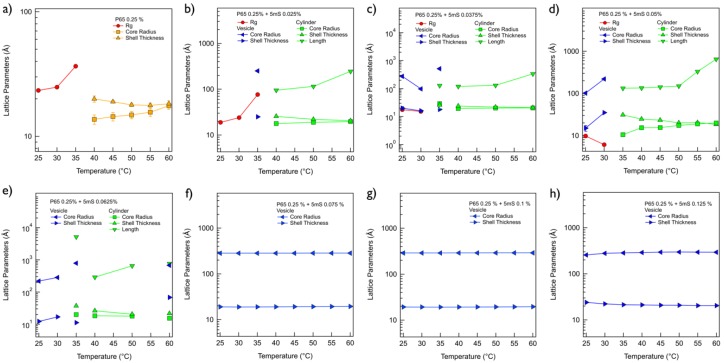
Fitting parameters obtained from the small angle neutron scattering (SANS) analyses of P65 (0.25%)-5mS (**a**) 0%, (**b**) 0.025%, (**c**) 0.0375%, (**d**) 0.05%, (**e**) 0.0625%, (**f**) 0.075%, (**g**) 0.1%, and (**h**) 0.125% mixtures.

Both P65 and P105 only solutions are very clear and transparent at room temperature. On the other hand, when the 5mS concentration increases, the P65-5mS and P105-5mS mixtures become cloudy with a bluish color at 0.0625% and 0.125% of 5mS concentrations, respectively, which comes from the Tyndall effect (Figure 1a,b). This may indicate that the large aggregates with several ten nanometers are formed in the cloudy solution. To confirm the size of the mixture solution depending on the 5mS concentration, the dynamic light scattering (DLS) measurements at 25 °C were carried out. Since the P65 at 0.25% does not form the micellar structure, the hydrodynamic diameter of the P65 only was not measured. While the hydrodynamic diameters of the P65-5mS mixture solutions with the low 5mS concentration (0.0375% and 0.05%) were ca. 23 nm, those above 0.05% were increased over twice. In the case of the P105-5mS mixture solution, the hydrodynamic diameters were drastically increased at the 5mS concentration of 0.125%, showing the Tyndall effect as well. These results are consistent with the inference from the visual inspection. Therefore, this indicates that the 5mS molecules combine with the Pluronic polymer and they contribute in the increase of the aggregate of Pluronic polymer, leading to various phase behaviors depending on 5mS concentration, even though the intrinsic nature in a molecular level of 5mS molecules binding with polymers is still unclear. It should be noted that the dramatic change of the hydrodynamic diameter and the observation of Tyndall effect for the P65-5mS mixture solution occurs in relatively low 5mS concentration when compared with the P105-5mS mixture solution. This arises from the difference of the molecular weight of Pluronic polymer because the P105-5mS mixture solution needs much more 5mS molecules to sufficiently modify the hydrophilic mass fraction of Pluronic P105 with larger PEO block than Pluronic P65.The detailed microstructures of the P65-5mS and P105-5mS mixture solutions depending on 5mS concentration have been investigated by small angle neutron scattering (SANS) experiments. In addition, to confirm the temperature dependency for the phase behavior of the P65-5mS and P105-5mS mixture, the temperature was controlled from 25 °C to 60 °C during SANS measurements. All of the mixture solutions for the SANS measurement were prepared in heavy water to enhance the neutron scattering contrast between the micro-structure and water, where their phase behaviors are visually identical to those that were prepared in water. The SANS intensities of the P65-5mS mixture solution at varying temperature are shown in Figure 2. The scattering intensity of Pluronic P65 only are fairly low in the whole *q* range below 40 °C (where *q* is the magnitude of the scattering vector (*q = (4π/λ)sin(θ/2)*), *θ* is the scattering angle), which is typical for a random polymer coil (Figure 2a). In fact, it is well known that Pluronic P65 polymer exists as the form of a random coil in aqueous solution around room temperature [31]. Therefore, the SANS intensity was analyzed by using the Debye model that is suitable for a randomly distributed polymer coil [22,23,24], and then was successfully reproduced with the radius of gyration (Rg) of Pluronic P65 polymer of 2.33~3.66 nm (Figure 3a). Above 40 °C, the SANS intensity of P65 only solution was very even in the low *q* region (*q* < 0.01 Å^−1^, which corresponds the Guinier region) and showed a *q*^−4^ behavior in the mid *q* region (0.04 Å^−1^ < *q* < 0.1 Å^−1^, which corresponds to the Porod region), indicating the formation of spherical shapes. Therefore, the core-shell spherical form factor [22,23,24] was adopted, which is modeled as the core region for PPO blocks and the shell region for PEO blocks. The core SLD was fixed as the value of Pluronic polymer, but the shell SLD, where water is able to penetrate into the PEO chain, was set to a free fitting variable. Then, the core-shell spherical form factor (the core radius of 1.37~1.83 nm and shell thickness of 1.76~1.99 nm) agrees with the SANS intensities well (Figure 3a). For the P65-5mS mixture with the 5mS concentration of 0.025%, the SANS intensity was also very low at 25 °C and 30 °C like the result of P65 only at 25~35 °C (Figure 2b), which was successfully reproduced by the Debye function with the Rg of 1.89 nm and 2.39 nm (Figure 3b). At 35 °C, the SANS intensity was quite increased, which is nearly *q*^−2^ behavior in the range of 0.005 Å^−1^ < *q* < 0.05 Å^−1^ indicating the layered structures such as lamellar or vesicular structures. However, the SANS intensity was not matched with the single layered form factor with any combination of fitting variable. Instead, a sum model with a vesicular form factor (which describes a shell SLD with Gaussian distribution) [25] and the Debye model was adopted and agrees well with the SANS intensity (where the vesicle core radius is 25.15 nm, the standard deviation of Gaussian distribution for the shell is 2.50 nm and the Rg is 7.65 nm. Interestingly, when the temperature further increases into above 40 °C, the SANS intensity entirely changed and then showed *q*^−1^ behavior (which is typical for a cylindrical shape) in the mid *q* region, instead of *q*^−2^ behavior, which indicates a topological phase transition. Here, the core-shell cylindrical form factor [22,23,24] was adopted to analyze the SANS intensity, which is modeled as the core region for PPO blocks and the shell region for PEO blocks and the SLDs of the core and shell region were set to the same as the core-shell spherical form factor. Using the core-shell cylindrical form factor, the SANS intensity at 40~60 °C was successfully reproduced with an appropriate fitting variable (core radius = 1.79~1.97 nm, shell thickness = 2.05~2.56 nm, and length = 9.50~24.53 nm, Figure 3b). For the P65-5mS mixture with the 5mS concentration of 0.0375~0.0625%, the SANS intensity is rather strong, even the room temperature, which comes from the nanosized inhomogeneity in the mixture solution, and it increases with temperature. At 25 °C and 30 °C, the SANS intensities could not be reproduced by the spherical and cylindrical form factor models with any combination of fitting variables. In contrast, the vesicular form factor model or a sum model of vesicular form factor and Debye model agrees very well with SANS intensities (Figure 2c–e), where the vesicle size ranges from ca. 20 nm to ca. 60 nm depending on the 5mS concentration and temperature (Figure 3c–e). Above 35 °C, the SANS intensity of the P65-5mS mixture with the 5mS concentration of 0.0375~0.0625% are very diverse depending on 5mS concentration (Figure 2c–e). The SANS intensity of P65-5mS mixture with the 5mS concentration of 0.0375% at 35 °C showed *q*^−2^ behavior in the mid *q* region while that above 40 °C indicated *q*^−1^ behavior in the mid *q* region, and then analyzed with a sum model of vesicular form factor and core-shell cylindrical models for 35 °C and a single core-shell cylindrical form factor model for above 40 °C. The fitted variables are shown in Figure 3c–e. In the case of the P65-5mS mixture with 5mS concentration of 0.05% above 35 °C, all of the SANS intensities showed *q*^−1^ behavior in the mid *q* region and were successfully reproduced by a core-shell cylindrical form factor model with appropriate fitting variables. As the temperature increases, the core radius and length of cylinders is increased from 1.05 nm to 1.99 nm and from 13.31 nm to 65.08 nm, respectively, but the shell thickness is decreased from 3.1 nm to 1.85 nm. This can be easily explained by the decrease of the effective hydrophilic mass fraction of Pluronic polymer arising from the increase of the hydrophobicity of Pluronic polymer upon heating. The SANS intensities of the P65-5mS mixture with 5mS concentration of 0.0625% above 35 °C are more complicated with an increasing temperature. In the range of 35~50 °C, the features of the SANS intensity were similar to that of the P65-5mS mixture with 5mS concentration of 0.0375%. However, at 60 °C, the SANS intensities of the P65-5mS mixture with 5mS concentration of 0.0625% showed *q*^−2^ behavior in the mid *q* region again. This complicated phase behavior is related to the hydrophobic interaction and the solubility of 5mS molecules depending on temperature, which is described in detail later. In the range of 5mS concentration from 0.075% to 0.125%, the SANS intensities of the P65-5mS mixtures were very simple, indicating *q*^−2^ behavior in the mid *q* region for whole temperature that was successfully reproduced by the vesicular form factor with nearly constant fitting variables (core radius = ca. 29 nm and shell thickness = ca. 2 nm) depending on temperature and 5mS concentration (Figure 3f–h). We expect that the core radius and shell thickness of the polymer vesicle can be controlled by the molecular weight of block copolymers. Since the membrane permeability of the vesicle structure for drug delivery is very crucial [8], it is very important to control the membrane thickness (which is directly related to the membrane permeability) for effectively transporting a variety of drugs into an organ. Therefore, we think that this study might provide a simple and easy way to control the membrane permeability of the vesicle structure.

**Figure 4:**
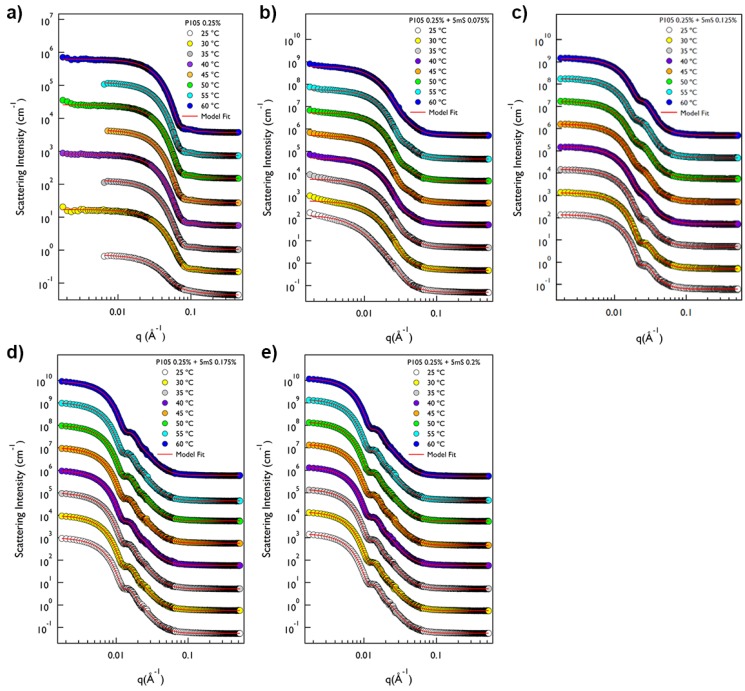
SANS intensities of P105-5mS mixtures at varying 5mS concentration (**a**) 0 wt%, (**b**) 0.075 wt%, (**c**) 0.125 wt%, (**d**) 0.175 wt%, and (**e**) 0.2 wt% and temperature.

**Figure 5:**
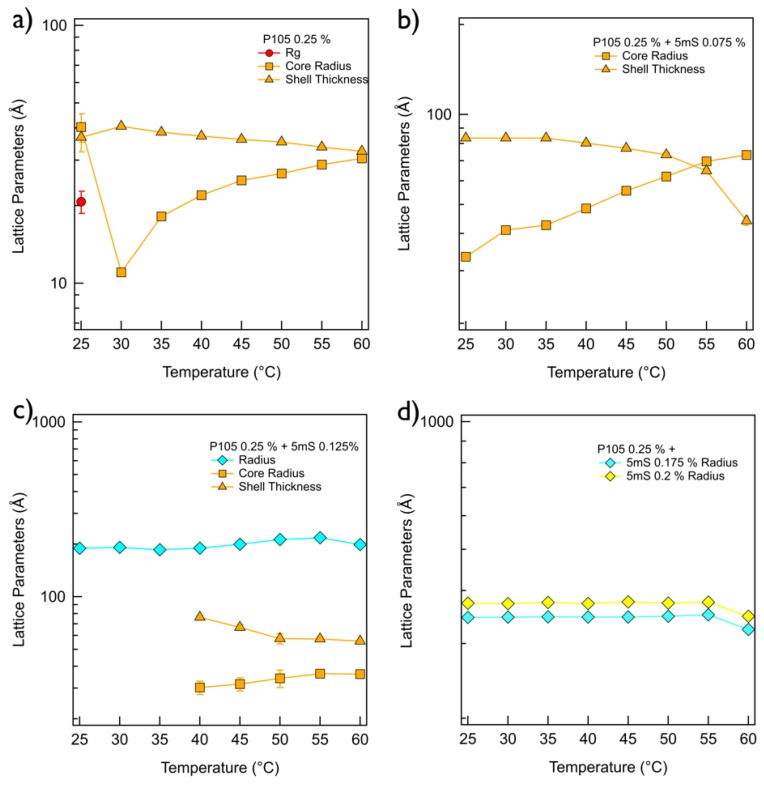
Fitting parameters obtained from the SANS analyses of P105 (0.25%)-5mS (**a**) 0 wt%, (**b**) 0.075 wt%, (**c**) 0.125 wt%, (**d**) 0.175 wt%, and (**e**) 0.2 wt% mixtures.

On the other hand, the phase behavior of the P105-5mS mixture is entirely different with that of P65-5mS mixture solution depending on temperature and 5mS concentration. The SANS intensity of P105 only solution at whole temperature was very flat in the low *q* region (*q* < 0.01 Å^−1^) and it showed a *q*^−4^ behavior in the mid *q* region (0.04 Å^−1^ < *q* < 0.1 Å^−1^), indicating the formation of spherical shapes (Figure 4a). The SANS intensity of the P105 only solution at 25 °C was analyzed by a sum model (which is effective to analyze the SANS data for two or three different kinds of particles) of the core-shell spherical form factor and Debye model with the fitting variables (core radius = 4.03 nm, shell thickness = 3.68 nm and Rg = 2.07 nm) (Figure 5a). The SANS intensity of the P105 only solution at 30~60 °C was well-fitted with a single model, the core-shell spherical form factor with the fitting variables (the core radius of 1.10~3.05 nm and shell thickness of 3.25~4.06 nm, Figure 5a). For the P105-5mS mixture solution with the 5mS concentration of 0.075%, the SANS intensity was not quite different with that of P105 only solution (Figure 4b), which is successfully reproduced by the core-shell spherical form factor with the fitting variables (the core radius of 3.34~7.32 nm and shell thickness of 4.41~8.35 nm, Figure 5b). As the temperature increases, the core radius of the P105-5mS mixture with the 5mS concentration of 0.075% is increased while the shell thickness is decreased, which arises from the increase of the hydrophobicity of PEO blocks. In addition, since the 5mS molecules can easily bind with Pluronic polymer with an amphiphilicity [29,30], it is expected to form a complex with relatively bigger size. Therefore, the overall size of the mixture that was obtained from the SANS analysis is rather bigger than that of P105 only at whole temperature. As the 5mS concentration further increases, the SANS intensity of the P105-5mS mixture solution is significantly changed, showing a clear bump in the mid *q* region (0.01 Å^−1^ < *q* < 0.03 Å^−1^), which may provide the size information of particle, and the left shift of Guinier region (Figure 4c–e). Considering the molecular weight of Pluronic P105 polymer and its possible micellar structure (where the hydrodynamic diameter of P105 polymer is ca. 10 nm, Figure 1b), being a bump of the SANS intensity in *q* < 0.03 Å^−1^ indicates that the formed structure is not a simple and small spherical particle but a bigger particle, such as vesicle or lamellae. However, the SANS intensity did not show *q*^−2^ behavior in the whole *q* region, which is typical for layered structures. Furthermore, with any combination of fitting variables for a single model with vesicle or lamellar structure, the SANS intensities were not reproduced. For the SANS data analysis, therefore, we adopted a single model with the spherical form factor with a big diameter or a sum model with spherical and vesicular form factors. For the P105-5mS mixture solution with the 5mS concentration of 0.125% at 25~35 °C, the SANS intensities were perfectly reproduced by the single spherical form factor with the whole radius of 18.6~19.2 nm, which is rather bigger than that of 0.075% (Figure 5c). It should be noticeable that the used spherical form factor is modeled as a simple sphere without a boundary between the core and the shell. Considering the molecular weight of polymer, it is difficult to form the core-shell spherical particle with a large diameter (>30 nm) without swelling the particle. To achieve the swelling in the entire particle, therefore, the water should be homogeneously distributed in the particle without a boundary of the core and the shell, and then the spherical form factor is enough to describe the spherical particle. Above 40 °C, since the single spherical form factor did not agree with the SANS intensity of the P105-5mS mixture at the bump (*q* ~ 0.02 Å^−1^), the fitting model was adjusted to a sum of the simple spherical and the core-shell spherical form factors that agrees with the SANS intensities very well. The fitting variables are shown in Figure 5c. The overall size of the spherical particle (where water is homogeneously distributed) (the radius of 20.0~21.8 nm) is much bigger than that of the core-shell sphere (the core radius of 3.02~3.62 nm and the shell thickness of 5.56~7.63 nm), which is comparable to the P105-5mS mixture with the 5mS concentration of 0.075%. The reason why the self-assembled structure of P105-5mS mixture (0.125% of 5mS concentration) forms two different spherical particles can be explained by the increase of the solubility of the 5mS molecules with temperature. Since the 5mS molecules are dissociated in water upon heating (due to the increase of the solubility of 5mS molecules), the amount of 5mS molecule in the P105-5mS mixture are relatively decreased, which leads to the formation of the core-shell spherical particle (which has the volume fraction of 23.7~42.6% depending on temperature), such as the P105-5mS mixture with below 0.075% of 5mS concentration. Interestingly, for the P105-5mS mixture solutions with the 5mS concentration of 0.175% and 0.2%, the SANS intensity was successfully reproduced by the single spherical form factor with the radius of 34.6~37.5 nm at whole temperature. This indicates that the P105-5mS mixture solution with the 5mS concentration of 0.175% and 0.2% is insensitive to temperature elevation, even though the spherical particle size is very unusual, where we expect that it is directly related to the origin of the phase behavior of Pluronic polymer arising from the control of the external conditions such as temperature and additives.

**Figure 6:**
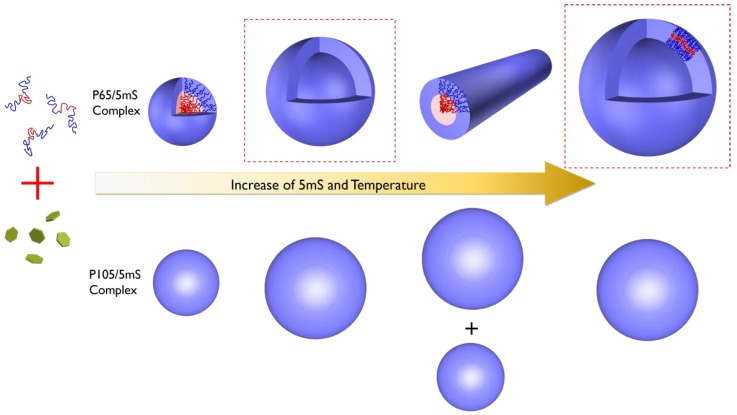
The schematics of the phase behavior of Pluronic polymer-5mS mixture depending on the 5mS concentration and temperature.

Based on the SANS analyses of the P65-5mS and P105-5mS mixture solutions, we confirmed the phase behavior of the polymer-additive complex depending the temperature, molecular weight of Pluronic polymer, and the concentration of additives. As the temperature and the 5mS concentration increases, the P65-5mS mixtures evolve from a random polymer coils into spherical particles, vesicles, cylindrical particles, and vesicles again, while the P105-5mS mixtures transform from a random polymer coils into spherical particles and bigger spherical particles, as shown in Figure 6.

The phase behavior of the Pluronic block copolymers-5mS molecule mixture solution can be understood by the coupled effect arising from the simultaneous increase of the hydrophobicity of Pluronic block copolymer and the solubility of 5mS molecules upon heating. Upon the increase of the hydrophobicity of block copolymers, leading to the decrease of the hydrophilic mass fraction of block polymer [7], it is natural that the Pluronic polymer-5mS molecule mixture self-assembles into spheres, cylinders, and layered structures, such as vesicles in consecutive order when the mixture forms the complex with a nanostructure. For the P65-5mS mixture solution, upon heating, we expect that the hydrophobicity of the mixture is increased by Pluronic polymer. In addition, since the 5mS molecule has the hydrophobic phenyl moiety, which has a significant portion in the 5mS molecule, the hydrophobicity of the mixture is also increased with the 5mS concentration, leading to the further decrease of the hydrophilic mass fraction. When considering the relatively small molecular weight of Pluronic P65, the self-assemblies of P65-5mS mixture are significantly affected by the temperature and 5mS concentration, and then they can be transformed from random coil polymers or spheres to vesicles, such as the P65-5mS mixture with 5mS concentration of 0.025% in temperature range of 25~35 °C (Figure 2a). However, since the solubility of 5mS molecules is highly dependent on the temperature, the 5mS molecules bound with Pluronic polymers are dissociated into the solvent with increasing temperature, leading to the relative increase of the hydrophilic mass fraction of the mixture. Therefore, the P65-5mS mixture solution with the 5mS concentration of 0.375~0.0625% shows the vesicle to cylinder phase transition between 30 °C and 35 °C (Figure 2c–e). As the temperature further increases, the cylinder structure changes to vesicle structure with a different size, arising from a relative increase of the hydrophobicity of block copolymer again. In the case of the P65-5mS mixture solutions with the 5mS concentration of 0.075~0.125%, they form the vesicular structure without the phase transition depending on temperature and 5mS concentration. Above 0.075% of the 5mS concentration, this indicates that the self-assembly of the mixture solutions is fully evolved into vesicular structure and the amount of 5mS molecules binding with Pluronic polymers is sufficient to maintain the vesicular structure even at 60 °C.

For the P105-5mS mixture solution, we expected the different phase behavior to the P65-5mS mixture. Since Pluronic P105 polymers have relatively larger molecular weight than the P65 polymer, much more 5mS molecules are required to induce a topological phase transition. Then, a lot of 5mS molecules with the limited solubility should be dissolved in the solvent, because the volume of the micellar structure is not enough to be fully bound with polymer for a topological phase transition, which is entropically unfavorable. Thus, the small size core-shell spherical particle might transform into simple spherical particles with a large size where water is homogeneously distributed, leading to the formation of the complex of all 5mS molecules and Pluronic polymers. The reason why two different size of spherical particle is formed may be similar to the origin of the vesicle to cylinder phase transition of the P65-5mS mixture (the dissociation of 5mS molecules from the complex upon heating).

## 4. Conclusions

We reported that the phase behavior of the mixture of the Pluronic P65 and P105 and organic derivative, 5-methyl salicylic acid (5mS) depending on the temperature and additives. Small angle neutron scattering measurements showed that the P65-5mS mixtures exhibited various self-assembled nanostructures, such as sphere, cylinder, and vesicle, while the P105-5mS mixtures showed spherical particles with two different sizes without any topological phase transition. In particular, the self-assembled structure of P65-5mS mixtures exhibited two different vesicles, depending on temperature, which can give possible applications in smart drug delivery system for an organ with specific temperature. Furthermore, the core radius and shell thickness of vesicle is variable depending on the molecular weight of block copolymers, which may provide the possibility for the permeability control of membrane against drugs. When considering the results of Pluronic triblock copolymer mixture (in the terms of the hydrophobicity of Pluronic polymer and the solubility of 5mS molecules) and two different Pluronic-5mS mixtures, the phase behavior of the Pluronic polymer-5mS molecule mixtures is highly dependent on the molecular weight of the polymer and temperature. This result can directly give fundamental information for the phase behavior of the polymer mixtures, which is very helpful for the practical use of Pluronic polymers in nano- and bio-science. Furthermore, this can provide a simple route for the fabrication of the nanostructure without a complicated procedure.
